# Border Disease Virus: An Exceptional Driver of Chamois Populations Among Other Threats

**DOI:** 10.3389/fmicb.2015.01307

**Published:** 2015-12-18

**Authors:** Emmanuel Serrano, Andreu Colom-Cadena, Emmanuelle Gilot-Fromont, Mathieu Garel, Oscar Cabezón, Roser Velarde, Laura Fernández-Sirera, Xavier Fernández-Aguilar, Rosa Rosell, Santiago Lavín, Ignasi Marco

**Affiliations:** ^1^Servei d’Ecopatologia de Fauna Salvatge, Departament de Medicina i Cirurgia Animals, Universitat Autònoma de BarcelonaBarcelona, Spain; ^2^Departamento de Biologia and Centro de Estudos do Ambiente e do Mar, Universidade de AveiroAveiro, Portugal; ^3^VetAgro-Sup, Université Claude Bernard Lyon 1Villeurbanne, France; ^4^Office National de la Chasse et de la Faune Sauvage, Unité Faune de MontagneGières, France; ^5^Institut de Recerca i Tecnologia Agroalimentàries-Centre de Recerca en Sanitat Animal, Universitat Autònoma de BarcelonaBarcelona, Spain; ^6^Ecole Nationale Veterinaire de ToulouseToulouse, France; ^7^Departament d’Agricultura, Ramaderia, Pesca Alimentació i Medi Natural, Generalitat de CatalunyaBarcelona, Spain

**Keywords:** emerging diseases, extinction risk, pestivirus, population viability analysis, keratoconjunctivitis, *Rupicapra*, sarcoptic mange, VORTEX

## Abstract

Though it is accepted that emerging infectious diseases are a threat to planet biodiversity, little information exists about their role as drivers of species extinction. Populations are also affected by natural catastrophes and other pathogens, making it difficult to estimate the particular impact of emerging infectious diseases. Border disease virus genogroup 4 (BDV-4) caused a previously unreported decrease in populations of Pyrenean chamois (*Rupicapra pyrenaica pyrenaica*) in Spain. Using a population viability analysis, we compared probabilities of extinction of a virtual chamois population affected by winter conditions, density dependence, keratoconjunctivitis, sarcoptic mange, and BD outbreaks. BD-affected populations showed double risk of becoming extinct in 50 years, confirming the exceptional ability of this virus to drive chamois populations.

## Introduction

In the early 21st century, infectious diseases are considered a substantial threat to planet biodiversity (Daszak et al., 2000). Habitat loss, overexploitation, invasive species, and climate change are the best known drivers of species extinction by far, in part due to the lack of information on the role of pathogens in species extinction (Smith et al., 2009). While the importance of pathogens in species conservation is common knowledge, few people understand the power of diseases to drive extinction, or cause important economic losses. An example of this potential role of pathogens occurred in the Central and Eastern Pyrenees in 2001, when a border disease virus was responsible for a dramatic decrease (over 80%) of several Pyrenean chamois (*Rupicapra pyrenaica pyrenaica*) populations (Marco et al., 2009). The etiological agent of these epidemics was classified into the Border disease virus genogroup 4 (BDV-4; Arnal et al., 2004), which had been present in the Pyrenees for at least two decades (Marco et al., 2011). The reasons for the emergence of the disease are still unclear (Marco et al., 2015). Pyrenean chamois is a flagship species that supports rural economies by attracting ecotourists and hunters from around the world. The epidemics caused a local cessation in game activities and the decline of hunting revenues. The regional administration was powerless in the face of the epidemics, and border disease gained notoriety among hunters, veterinarians, wildlife biologists, and the local population. Strict regulations shut down hunting in areas showing mortality or low fertility or limited hunting bags in game states with the presence of the virus. While these measures aimed to minimize chamois mortality in the Pyrenees, the effects of the epidemics remain (Marco et al., 2015). However, it is unclear if the attention paid to BDV is groundless given that other older diseases (e.g., infectious keratoconjunctivitis, IKC or sarcoptic mange, SM) have also caused population collapses of chamois throughout Europe. In this perspective article, we aim to elucidate this question of whether BDV does in fact threaten the population viability of Pyrenean chamois, and whether its impact is more important than the risk associated with other/previous epidemics. We used a stochastic simulation of the risk of extinction in a fictitious chamois population regulated by density dependent processes, climate events, and the effect of old and emerging infectious disease, namely border disease (BD). Moreover, we briefly review the natural history of the affected host (*Rupicapra* spp.) and the epidemiology of these three diseases.

## A Short Biosketch of Chamois

Pyrenean chamois (*R. p. pyrenaica*) is by far the species most vulnerable to BDV infection. The northern chamois (*R. rupicapra*), however, has been suggested to act as a spillover, but no outbreak has been recorded to date (Martin et al., 2011; Fernández-Sirera et al., 2012b). This short biosketch summarizes the details of both species that are determinant for our modelling purposes.

*Rupicapra* is a long-lived (life expectancy 21 years; Gonzalez and Crampe, 2001), medium-sized, mountain-dwelling mammal inhabiting central and southern Europe. These caprinae species are nearly monomorphic with males about 20–33% larger than females (Pépin et al., 1996; Garel et al., 2009; Rughetti and Festa-Bianchet, 2011). This mammal follows a capital breeder strategy showing compensatory feeding in advance of breeding attempts (Houston et al., 2007). In fact, males gain much more mass (40% heavier) than females from spring to autumn in anticipation of the rutting period (November–December), and this difference decreases reaching a minimum in early spring (4%, Rughetti and Festa-Bianchet, 2011). Female chamois are basically monotocous (170 days gestation period, 1 offspring per year, and rarely twins) with a moderate degree of polygyny (Loison et al., 1999b), e.g., about four females for a given male and year (Corlatti et al., 2013). Though the female chamois is sexually mature at 18 months of age (Couturier, 1938), it rarely contributes to population demography before 3 years of age. In colonizing populations, two-year-old females can contribute significantly to recruitment (63.3–95%, Houssin et al., 1993; Loison et al., 2002). As the density increases, age at primiparity shifts from two to three years old with a proportion of reproducing females varying from 80% (Storch, 1989) to more than 90% (Houssin et al., 1993; Pérez-Barbería et al., 1998; Loison et al., 2002). Overall, in early summer more than 80% of prime-aged females (3–8 years) are accompanied by a kid (Houssin et al., 1993; Pérez-Barbería et al., 1998; Loison et al., 2002).

In females, reproductive success is stable until at least 10 or 14 years of age but begins to decrease between 12 and 16 years of age (Crampe et al., 2006; Tettamanti et al., 2015). In males, reproductive success has not been properly assessed, but field observations suggest that only fully adult males (≥6 years) hold the largest harems and copulate most often (Lovari and Locati, 1991; Corlatti et al., 2013, 2015). Thus, longevity appears to be the main determinant of lifetime reproductive success in chamois. There is a slight but detectable cost of reproduction in males during the mating season (28% decrease in body mass; Mason et al., 2011) that is unappreciable in females (Garel et al., 2011a). Recruitment rates (proportion of offspring surviving through the winter per female) are lower for young (3–4 years) females (0.15–0.22) than for prime-aged females (0.41 per year for 5- to 16-year-old females, Crampe et al., 2006). Generation time varies from 5 to 8 years (Crampe et al., 2006).

The annual survival rate is normally low in kids (<1-year-old, 58%; Loison et al., 1994) and high in maturing (91%, for 1.5–3.5 years old) and adult individuals (96%; Loison et al., 1999a; Gonzalez and Crampe, 2001; Corlatti et al., 2012). Mortality of kids (<1 year) is higher (42%) and fluctuates more than in the other age classes (Crampe et al., 2002). Interestingly, there are no sexual differences in mortality rates (Loison et al., 1999a; Gonzalez and Crampe, 2001; Bocci et al., 2010; Corlatti et al., 2012).

## Density Dependence

The growth rate of chamois populations is affected by density at a time lag of 1 year (Willisch et al., 2013), i.e., animal numbers in 1 year negatively influence population growth in the following year. The fertility rate (kid/female ratio) is the main trait affected by delayed density dependence. Other authors (Capurro et al., 1997) observed that such delayed effects of density (2-year lag) did not affect birth rates but rather that total mortality rates of both kids and adults increased by up to 72% or 19–21%, respectively.

## Environmental Dependence

The role of exceptional snowy winters as stochastic factors regulating chamois populations (Schröder, 1971) has long been recognized. Seasonal snow cover limits locomotion and access to forage, and low temperatures increase thermoregulatory cost. Though the behavior of chamois aims to compensate for food shortages caused by wintertime, prolonged snow cover, and avalanches shape chamois populations (Jonas et al., 2008). In fact severe snowfalls (e.g., 165–590 cm of cumulative snowfall) can increase mortality by more than twice that recorded in normal winters (Crampe et al., 2002; Rughetti et al., 2011). The impact of extreme snow falls is especially severe for kids (Willisch et al., 2013) and adult age classes (>10 years; Rughetti et al., 2011), without a strong impact on reproduction. Though winter cumulative snowfalls in these ecosystems show great interannual variation, episodes of heavy snow falls affecting chamois populations tend to occur at least once every 10 years (Capurro et al., 1997; Rughetti et al., 2011; Willisch et al., 2013). In addition, early summer conditions determine chamois population dynamics through their effect on diet quality (Gálvez-Cerón et al., 2013; Villamuelas et al., 2015), body growth, reproductive success, and survival (Garel et al., 2011b).

## The Impact of Old Infectious Diseases

The influence of diseases on chamois populations had been reported by the early 20th century. IKC and SM are two of the best known infectious diseases with relevance for the viability of chamois populations.

IKC caused by *Mycoplasma conjunctivae* affects domestic and wild caprinae worldwide (Giacometti et al., 2002). The infection produces unilateral or bilateral inflammation of the conjunctiva and in advanced stages results in corneal opacity and transient blindness. Recovery from the disease is possible, but the ocular lesion may progress to corneal ulceration and perforation, or a non-healing lesion that leads to death due to starvation or accident. Consequently, the impact of IKC in populations of chamois is often critical. In the wild, the number of sick individuals peaks in summer (Loison et al., 1996; Arnal et al., 2013) since flies are suspected to contribute to spread the disease (Giacometti et al., 2002). The first reported outbreak of IKC in wild ungulates, chamois in the Austrian Alps, dates to 1916. Since then, IKC outbreaks are commonly reported in chamois populations from the Alps and Pyrenees (Giacometti et al., 2002; Arnal et al., 2013). These IKC outbreaks are characterized by a short duration of 1–2 years (Loison et al., 1996; Arnal et al., 2013), high morbidity, low mortality, and spontaneous recovery (Loison et al., 1996). In fact, individuals that overcome the infection can show lower infection susceptibility in subsequent epizootic episodes. However, IKC is sometimes associated with high mortalities (>30%; Loison et al., 1996; Giacometti et al., 2002), with the reasons for extreme events largely unknown.

Females and juveniles are especially affected by IKC with the number of affected adult males usually being low (Arnal et al., 2013). Sexual segregation between males and females during the summer could be related to this sex-biased susceptibility. In other cases, the age-class distribution of cases attributable to an IKC outbreak appears proportional to the initial population structure (Arnal et al., 2013). After an IKC epizootic episode, fertility of female chamois (number of kids/adult females) experiences a slight decrease (10–19%; Loison et al., 1996; Arnal et al., 2013) and begins to recover 1 year after the outbreak. This decline in reproductive index during the early post-epidemic periods may have resulted from a low neonatal survival. Occasionally IKC infection in chamois becomes endemic and outbreaks with mild consequences are observed every 3–4 years (Gauthier, 1994).

SM epizootics caused by the burrowing mite *Sarcoptes scabiei* also have a recognized impact on wildlife conservation (Pence and Ueckermann, 2002). Infected animals typically suffer from severe dermatitis, becoming dehydrated, emaciated and eventually dying from the infection. Amongst caprinae hosts, scabies-induced mortality of chamois populations has been reported for slightly over a century in the Alps (Onderscheka, 1982; Rossi et al., 1995), and for more than a decade in the Cantabrian Mountains, northwestern Spain (Fernández-Morán et al., 1997). No sex or age class has been shown to have higher susceptibility to scabies, and the potential effect of SM on either fertility or recruitment of females in diseased populations has not been determined. The number of chamois with visible scabies lesions peaks from late winter (March; Rossi et al., 2007) to late spring (May; Fernández-Morán et al., 1997). Rare cases are observed in summer and autumn. Demographic decline due to SM is highly variable. The epidemic cycle is characterized by an initial peak of infection associated with high mortality (>80%) followed by successive epidemic waves with lower impact (10–25%; Lunelli, 2010). Though the initial growth rates of some populations recover 2 years after the initial outbreak (Fernández-Morán et al., 1997), the impact of SM peaks from 4 to 6 years after the first scabietic animals are observed (Rossi et al., 2007; Turchetto et al., 2014).

Pneumonia caused by Pasteurellaceae species (e.g., *Mannheimia haemolytica, M. glucosidal*, *or Bibersteinia trehalosi*), *Mycoplasma* spp. or respiratory viruses are another cause of acute die-off of chamois populations (Citterio et al., 2003; Posautz et al., 2014). Unfortunately, demographic data describing the impact of pneumonia outbreaks on chamois populations is scarce and incomplete. Thus, this polymicrobial disease was not included in our population viability analysis.

## BDV: A Key Population Driver

Border disease virus belongs to the Pestivirus genus (Flaviridae family), is distributed worldwide and can cross the species barrier. The virus can be transmitted horizontally, by direct contact, and vertically *in utero* resulting in abortion of the fetus or in the birth of a persistently infected (PI) individual, depending on the period of gestation, with a short life expectancy (Schweizer and Peterhans, 2014). BD causes important economic losses on farms and virulent strains can cause systematic reproductive failure (Nettleton et al., 1999) and high mortalities in sheep (Chappuis et al., 1986; Vega et al., 2015).

In chamois, BD infection has severe consequences causing mortality in individuals of all ages, being considered an emerging disease for chamois populations in the Pyrenees. Clinical signs in naturally infected chamois include emaciation, alopecia, and neurological depression, the latest associated with non-suppurative encephalitis (Marco et al., 2007). Abortion has been also described under experimental conditions (Martin et al., 2013). Mortality rates vary enormously among populations (Fernández-Sirera et al., 2012a). In fact, while most populations are severely affected by successive outbreaks, others appear to coexist with the virus without consequence (Marco et al., 2015). An age-structured dependent infection (Pioz et al., 2007) and a seasonal spread of the virus (Beaunée et al., 2015) have been suggested for chamois populations. Viral mutation, host factors, climatic variation, and other ecological conditions may be playing an unknown but important role in explaining these different epidemiological scenarios.

## Stochastic Simulation of Population Extinction

An assessment of the risk of extinction is often required for conservation and management plans. The most realistic models incorporate causes of fluctuations in population size to predict probabilities of extinction (Boyce, 1992). In fact, many life history traits are in essence stochastic. Population viability analysis (PVA) is a method of quantitative analysis to determine the probability of extinction of a given population (Boyce, 1992). VORTEX (Lacy, 1993) is a powerful software for stochastic simulation of the extinction process under a broad range of situations (e.g., harvesting rates, age-specific reproduction rates, fixed or random catastrophes, and among others).

## Basic Simulation Input

We used VORTEX 10.0.7.0 (Lacy et al., 2015) to estimate viability of a population of an initial size of 500 chamois in a hypothetical scenario with a carrying capacity of 4000 individuals. Extinction was reached when population numbers were reduced to 30 individuals, the minimum viable population size recommended for a successful reintroduction of chamois (Lovari et al., 2010). Each population was simulated for 50 years and 1000 iterations. Neither inbreeding depression (i.e., reduction of a first-year survival among inbred individuals) nor concordance of reproduction and survival were considered in our PVA. Though dispersal has been reported in chamois populations (Loison et al., 1999c; Crampe et al., 2007), our theoretical population was considered closed. Data on the reproductive system, reproductive rates, and mortality rates are summarized in **Table 1**. Concerning catastrophes, our population had a yearly probability of 0.1 of experiencing an exceptionally snowy winter (at least five heavy snow falls over the study period). The impact of this extreme environmental variation on chamois survival is summarized in **Table 1**. No impacts on reproduction are expected after such catastrophes, but after a severe winter the carrying capacity of the ecosystem will increase by 10%. In fact, after severely snowy winters the availability of nutritious plants in the Alpine pastures increases the following summer (Pettorelli et al., 2005), favoring body weight gains and hence survival (Garel et al., 2011b). Finally, although we did not consider density-dependence effects on reproduction rates of adult females, the potential effects of overcrowding on mortality of young age classes was considered to be increasing by 10% of the standard deviation of mortality rates.

**Table 1:** Summary of parameter input base values used in the population viability analysis (PVA) of Chamois (*Rupicapra*).

Simulation input	Base value	Source
**Reproductive system and rates**		
Breeding system	Polygyny	Loison et al., 1999b
Age of first reproducing females	2–3	Loison et al., 2002
Age of first reproducing males	5–6	Corlatti et al., 2013, 2015
Maximum age of female reproduction	16	Crampe et al., 2006
Maximum age of male reproduction	16	NA
Maximum number of litter per year	1	Loison et al., 1999c
Maximum number of young per year	1	
Sex ratio at birth	1:1	Crampe et al., 2006
Breeding at low density (%)	70 for 2 years old females 90 for ≥3 years old females	Houssin et al., 1993; Pérez-Barbería et al., 1998; Loison et al., 2002
% Adult females breeding	88 (7)	Couturier, 1938; Storch, 1989; Houssin et al., 1993; Pérez-Barbería et al., 1998; Loison et al., 2002


% Adult females having one litter per year	100	


Average litter size	1	
Maximum litter size	1	
**Mortality rates**		
% Mortality from age 0–1 years	42 (37)	Loison et al., 1999a; Crampe et al., 2002; Loison et al., 2002; Rughetti et al., 2011


% Mortality from age 1–2 years	19 (17)	
% Mortality from age 3–10 years	18 (17)	
% Mortality for 10 years old	74 (28)	
**Catastrophes**		
Severe snow fall	At least once a decade	Crampe et al., 2002; Jonas et al., 2008; Rughetti et al., 2011
**Mortality rates due to disease outbreak**	
Keratoconjunctivitis outbreak (2 years)	6% kids (13) and 70% yearlings (18), 20% females (13) and 9% males (9)	Loison et al., 1996; Giacometti et al., 2002; Arnal et al., 2013
Sarcoptic mange outbreak (5 years)	10.5% kids (18) and 14% yearlings (6.5), 52.5% females (26.5) and 60% males (18)	Rossi et al., 1995, 2007; Fernández-Morán et al., 1997
Border disease outbreak (5 years)	50.5% kids (58.5), 51.8% yearlings (75.7), 45.7% females (86.8), and 47% males (19.5)	Marco et al., 2007, 2009; Fernández-Sirera et al., 2012a; Annual counts of the Catalan Department of Agriculture, Livestock, Game, Fishery, and Food

*The numbers in the column “Source” correspond to the references used for a given base value. Data in parenthesis is the standard deviation of the mean base value due to environmental variation. We assume no sexual differences in mortality rates. NA indicates no information available*.

## Disease Simulation Input

We used a PVA to compare the impact of IKC and SM outbreaks with the effect of BD epidemics on stochastic population growth rate (*r*), mean population size (*N*), standard deviations (*SD*_r_, *SD*_N_) and confidence intervals (95%) of a simulated population of chamois. We modeled four populations: one pathogen-free, a second affected by IKC, another by SM and the last by BD. The length of the epidemics was estimated by averaging the mean number of years that chamois population is affected by the disease after the first outbreak (i.e., clinical cases are detected and/ or population parameters differ from the pre-epidemic period): 2 years for IKC (Loison et al., 1996; Giacometti et al., 2002; Arnal et al., 2013), 5 years for SM (Fernández-Morán et al., 1997; Rossi et al., 2007), and 5 years for BD (Fernández-Sirera et al., 2012a).

The effect of diseases on the host was modeled as sex and age-specific harvesting rates (i.e., extra increase in chamois mortality during the epidemic). To compare probability of extinctions due to the effect of diseases, we ran 60 simulations (20 for each pathogen) with outbreaks occurring in different population sizes (from 600 to 1550, about 50 each). Results were compared by ANOVA and a Tukey’s HSD *post hoc* test. Details about our scenario settings are summarized in **Table 1**.

## The Good, the Bad, and the Ugly

**FIGURE 1:**
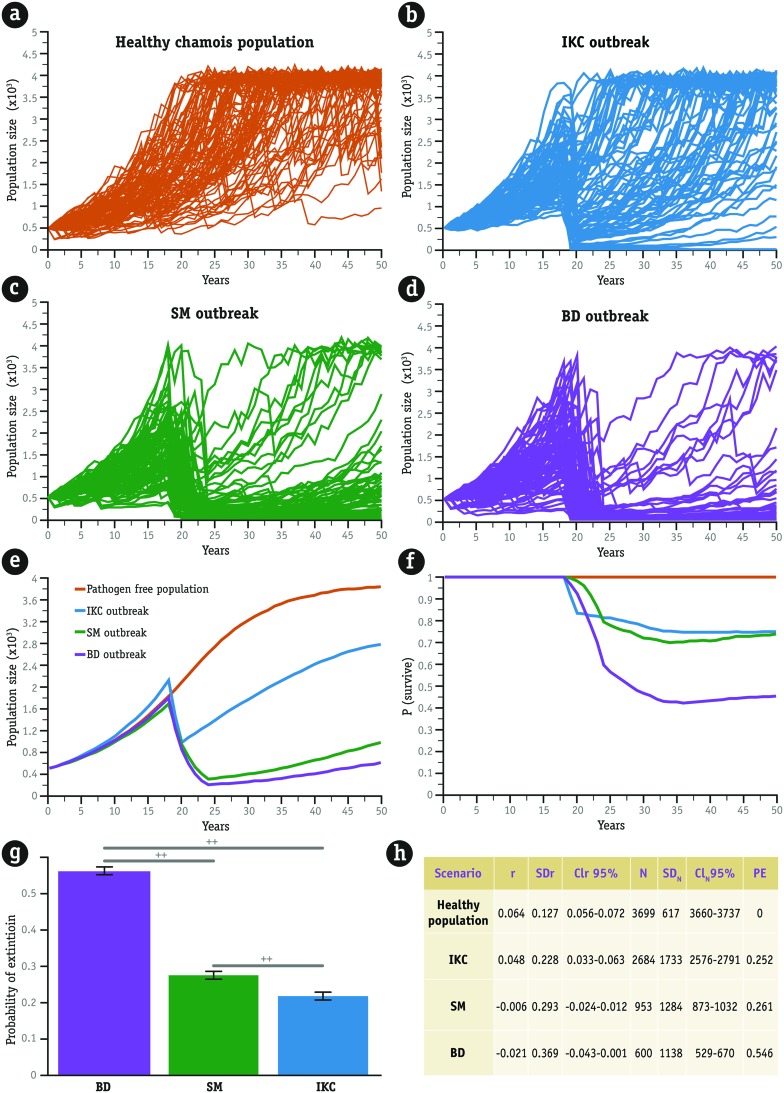
**Stochastic simulations of the extinction process in a fictitious population of 500 chamois for 50 years**. The hypothetical scenario has a carrying capacity of 4000 individuals and suffered the demographic consequences of five heavy winters. **(a)** A pathogen-free population of chamois only driven by density dependence and climate severity. **(b)** The consequences of a severe 2-year outbreak of infectious keratoconjunctivitis (IKC) in year 20. **(c,d)** The simulation of 5-year sarcoptic mange (SM) and border disease (BD) outbreaks, respectively, are shown, also in year 20. The number of populations that went extinct, represented by lines perpendicular to the *X* axis in **(b–d)** is greater for the population affected by BDV than for the other two (summarized in **e**). In any case, extinction was reached when the population number was reduced to 30 individuals. We performed 1000 simulations in each case, but **(a–d)** plots show only the output of the first 100 simulations. Along the same line, **(f)** shows the lower probability of survival after a disease outbreak. Information presented in plots **(e)** and **(f)** is based on 1000 simulations. The bar plot in **(g)** summarizes the results of ANOVA aimed at testing differences between probabilities of extinction over 50 years caused by the three diseases. These probabilities were calculated for 60 simulations (20 for each pathogen), with outbreaks occurring at different population sizes (from 600 to 1550, 50 simulations for each case). Whiskers represent the standard deviation and the horizontal lines the results of a *post hoc* Tukey’s HSD test. Statistically significant differences, at α = 0.05, are indicated by crosses. Statistical summary is shown in table **(h)**. Mean stochastic growth rate of the population (*r*), Mean final population size (*N*), associated standard deviations (*SD*_r_, *SD*_N_) and confidence intervals at 95% (CI_r_, CI_N_), and mean probability of extinction (PE) after our 1000 simulations.

Despite having suffered the consequences of five severe winters, our pathogen-free population of chamois grew from 500 to 3699 chamois in 50 years (**Figures 1a,h**). Consequently, the stochastic growth rate was positive (Stoch-*r* = 0.064, **Figure 1h**) and no extinction process occurred during the simulation. However, growth rate of the healthy population and hence the probability of extinction was seriously affected by the three infectious agents (*F*_2,27_ = 358.8, *p* < 0.01, **Figures 1f,g**).

Summarizing the impact of the three pathogens and inspired by the prominent film of the master Sergio Leone, we can imagine that IKC (**Figure 1b**) plays the “good” character, SM the “ugly” (**Figure 1c**), with the border disease virus surely cast in the role of the “bad” (**Figure 1d**, but see **Figures 1e,f** for a multiple comparison). After our simulated outbreaks, growth rate of the affected population decreased from 0.048 (IKC) to -0.021 units (BD), whereas probability of extinction ranged between 0.25 (IKC) and 0.55 (BD). Probability of extinction for a healthy population was 0 (**Figure 1h**, table). Though the impact of SM was between IKC and BD, the probability of extinction caused by each pathogen was statistically different (**Figure 1**, Tukey’s HSD test at α = 0.05). It is interesting to note that the relative standard deviation of the mean extant populations was greater after the BD outbreak (100 × *SD*/Mean = 189%) than after the other outbreaks (134% for the SM outbreak and 64% for IKC; **Table 1**). This result agrees with the variety of epidemiological scenarios of BD in the populations of Pyrenean chamois (Fernández-Sirera et al., 2012b)

## Concluding Remarks

Though our population modeling is not free of limitations (e.g., assumes a fixed length for the epizootics, only one epizootic at a time, no previous hunting-harvesting, lack if recovery due to herd immunity), it is able to illustrate quite well the impact of BD on the population dynamics of chamois. The IKC is characterized by short (1–2 years) and female-biased outbreaks (Arnal et al., 2013), affecting young age classes more, whereas SM shows longer outbreaks (from 2 to 6 years; Rossi et al., 2007), affecting all age and sex classes. BD epidemics, however, are the only of the three resulting in long outbreaks (>10 years in some populations) with abortions, neonatal and adult mortality. Moreover, the existence of persistent infected individuals can lead to the circulation of BDV among individuals over long periods of time which is an exceptional peculiarity of this pathogen. That peculiarity has at least been proven in domestic flocks (Schweizer and Peterhans, 2014), and in theoretical models for BD epidemics in chamois populations (Beaunée et al., 2015). To date, however, there are few evidences for the existence of PI in chamois populations (Marco et al., 2015). Alternatively, we cannot rule out the possibility of chronic shedding (non-PI individuals by definition) for explaining viral maintenance in chamois (Cabezón et al., 2011; Martin et al., 2013). In any case, both mechanisms (PI and/or long-lasting viraemia) would contribute for viral persistence of BDV in chamois populations.

Comparing the impact of several diseases from field data is not straightforward, as epidemics occur in different populations, at different stages of colonization and in contrasting environments. The simulation approach allowed us to compare the impact of the three studied pathogens considering similar situations and taking into account stochastic processes. Among the three pathogens under study, BDV showed the highest probability of extinction over 50 years: this probability reached values >50%, and thus even large host populations may go extinct under the pressure of intense epidemics. Given this high probability of host extinction, we argue that BDV is an exceptional driver of chamois populations and entails specific extinction risk. Further research should be oriented to illustrate more realistic scenarios, e.g., combining the impacts of more than one epidemics for a given period (IKC + BD) or including the impact of hunting-harvesting. Management actions designed to limit the impact of the virus should be evaluated and implemented, as the natural host-pathogen dynamics may not reach equilibrium in a near future.

## Author Contributions

AC-C and ES performed the literature review. ES analyzed the data. All authors contributed to conceiving the project and participated in the interpretation of results and final preparation of the paper.

## Conflict of Interest Statement

The authors declare that the research was conducted in the absence of any commercial or financial relationships that could be construed as a potential conflict of interest.
